# Perspective: APOBEC mutagenesis in drug resistance and immune escape in HIV and cancer evolution

**DOI:** 10.1093/annonc/mdy003

**Published:** 2018-01-08

**Authors:** S Venkatesan, R Rosenthal, N Kanu, N McGranahan, J Bartek, S A Quezada, J Hare, R S Harris, C Swanton

**Affiliations:** 1 CRUK Lung Cancer Centre of Excellence, UCL Cancer Institute, London, UK; 2 Translational Cancer Therapeutics Laboratory, The Francis Crick Institute, London, UK; 3 Danish Cancer Society Research Center, Copenhagen, Denmark, UK; 4 Science for Life Laboratory, Stockholm, Sweden; 5 Division of Genome Biology, Department of Biochemistry and Biophysics, Karolinska Institute, Stockholm, Sweden; 6 Cancer Immunology Unit, UCL Cancer Institute, London, UK; 7 International AIDS Vaccine Initiative (IAVI), New York, USA; 8 Masonic Cancer Center, Minneapolis, USA; 9 Institute for Molecular Virology, Minneapolis, USA; 10 Center for Genome Engineering, Minneapolis, USA; 11 Department of Biochemistry, Molecular Biology and Biophysics, University of Minnesota, Minneapolis, USA; 12 Howard Hughes Medical Institute, University of Minnesota, Minneapolis, USA

**Keywords:** APOBEC, immune escape, drug resistance, human immunodeficiency virus, intratumour heterogeneity

## Abstract

The apolipoprotein B mRNA-editing enzyme, catalytic polypeptide-like (APOBEC) mutational signature has only recently been detected in a multitude of cancers through next-generation sequencing. In contrast, APOBEC has been a focus of virology research for over a decade. Many lessons learnt regarding APOBEC within virology are likely to be applicable to cancer. In this review, we explore the parallels between the role of APOBEC enzymes in HIV and cancer evolution. We discuss data supporting the role of APOBEC mutagenesis in creating HIV genome heterogeneity, drug resistance, and immune escape variants. We hypothesize similar functions of APOBEC will also hold true in cancer.

Key Message

APOBEC mutagenesis creates diversity within viral and tumour cell populations. Sublethal APOBEC mutagenesis underlies the formation of HIV drug resistant and immune escape variants, and similar functions for APOBEC in cancer are expected. Incomplete inhibition may promote escape whereas, complete APOBEC inhibition may be attractive as adjuvant therapy.

## Introduction

Apolipoprotein B mRNA-editing enzyme, catalytic polypeptide-like 3 (APOBEC3; A3) is the name of a seven-membered family of single-stranded DNA cytosine deaminases in humans. Independent approaches including analyses of next-generation sequencing data implicated APOBEC-catalysed DNA damage and mutagenesis in breast cancer [1, 2]. Subsequent studies confirmed and extended the involvement of APOBEC in mutating the cancer genome to at least 16 other cancer types [3–5]. APOBEC signature mutations (C-to-T and C-to-G in TCA and TCT trinucleotide motifs) are the most prevalent in cancer after those attributable to ageing (C-to-T in CG dinucleotide motifs, most likely due to water-mediated deamination of methyl-cytosine) [4]. Furthermore, the clinical relevance of APOBEC in cancer is underscored by associations with poor patient outcomes and treatment resistance [6, 7], activation of oncogenic drivers [8–10], tumour subclonal diversification [9, 11, 12], and increased prevalence in metastases in comparison with primary tumours [13].

Although involvement of APOBEC mutagenesis in cancer has only recently come to light, these enzymes have been a focus of virology research for over a decade, beginning with the near simultaneous discoveries of APOBEC3G (A3G) as an HIV-1 restriction factor and as a DNA cytosine deaminase [14, 15] (reviewed elsewhere [16, 17]). We envision that many lessons learnt regarding APOBEC within virology will be applicable to oncology. For this reason, we explore the parallels between the role of APOBEC in HIV and cancer mutagenesis. We will especially focus on how APOBEC mutagenesis can promote intratumour heterogeneity, drug resistance, and immune escape.

## The AID/APOBEC superfamily: a diverse set of cytosine deaminase enzymes implicated in cancer

APOBEC3 belongs to the AID/APOBEC superfamily, consisting of activation induced deaminase (AID), APOBEC1 (A1), APOBEC2 (A2), APOBEC3A-H (A3A, A3B, A3C, A3D, A3F, A3G, and A3H), and APOBEC4 (A4). AID deaminates cytosines at the immunoglobulin locus, enabling antibody gene diversification via somatic hypermutation, and class switch recombination [18]. A1 was identified originally as an RNA editing enzyme, deaminating apolipoprotein B mRNA at a specific position to create an early stop codon [19], but it also has robust DNA deamination activity [14, 20]. The functions of A2 and A4 are still unclear and these proteins have yet to show enzymatic activity.

In general, the A3 family members are considered part of the innate immune system, forming overlapping barriers to virus and transposon replication. Consistent with such a physiological function, *A3* genes show profound copy number and amino acid variation in mammals. For instance, most humans have seven *A3* genes arranged in tandem, whereas rodents have only one at the same genomic location [21, 22], and each *A3* gene in humans as well as several other mammals manifests high levels of amino acid variation due to positive selection [23].

A3G has been studied intensely in the field of virology, as it was recognized early on to deaminate cytosines in cDNA reverse transcription intermediates of retroviruses including HIV-1 [24, 25]. Reverse transcriptase places an adenine opposite to the newly created uracil nucleobase, introducing a viral genomic strand G→A mutation [26]. This inhibits HIV replication by directly rendering the viral genome dysfunctional or by indirectly triggering viral cDNA degradation by subsequent uracil DNA glycosylase activity and endonuclease digestion [26, 27]. A3G can also directly bind to HIV-1 genomic RNA and interfere with viral cDNA synthesis [28]. A3D, A3F, and A3H also contribute to HIV-1 mutagenesis through similar mechanisms, and it is generally accepted that different subsets of A3 family members restrict the replication of different classes of viruses and transposons (reviewed elsewhere [16, 17]).

Adding to the complexity of seven A3 family members in humans, different subsets of A3 enzymes are expressed in different tissue types [29, 30]. Together with high levels of DNA sequence similarity (near perfect identity in many regions), determining which of these enzymes is responsible for mutagenesis of different cancer genomes has been challenging. Thus far, the greatest numbers of publications support A3B and A3A (reviewed more extensively elsewhere [31–33]). However, recent reports indicate that A3H is also important and, together with A3B, may account for the entire APOBEC mutation signature observed in breast and lung cancers [34, 35].

## APOBEC mutagenesis increases subclonal diversity

**Figure 1.**
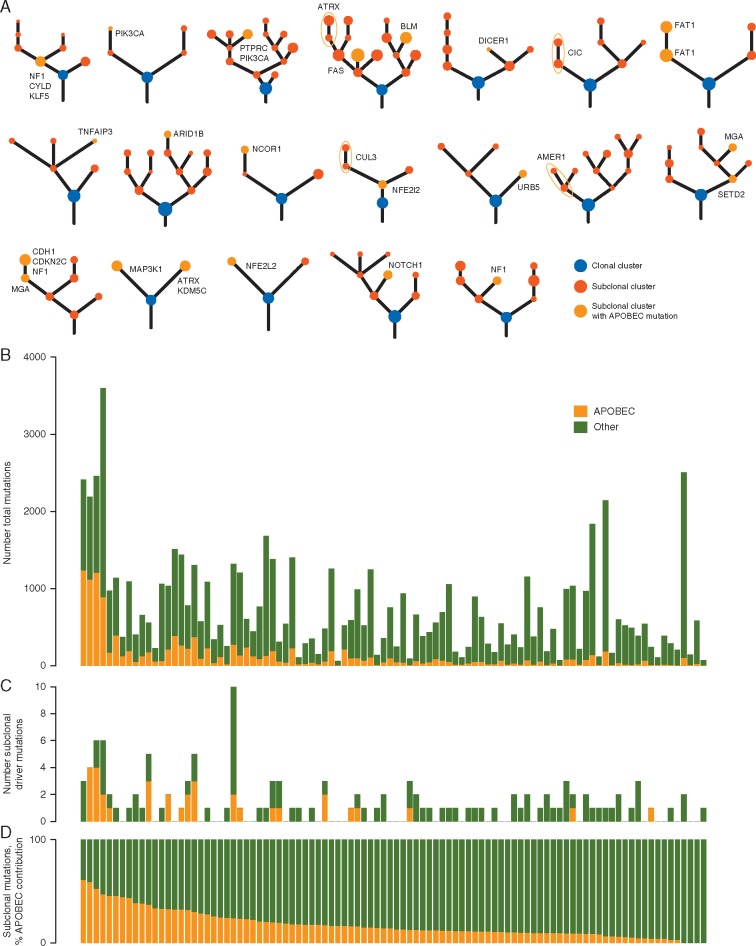
Apolipoprotein B mRNA-editing enzyme, catalytic polypeptide-like (APOBEC) mutagenesis within the TRACERx 100 cohort. (A) Phylogenetic trees of TRACERx patients harbouring a subclonal driver mutation in an APOBEC preferred motif are shown. The mutation is indicated near the clone in which it occurs. Clonal clusters are shown in blue, subclonal clusters are shown in red, and subclonal clusters containing the driver mutation in a preferred APOBEC motif are shown in orange. (B) The total numbers of mutations in each patient are shown, with mutations in an APOBEC context shown in orange and all other mutational contexts shown in green. (C) The total number of driver mutations in each patient are shown, with mutations in an APOBEC context shown in orange and all other mutational contexts shown in green. (D) The fraction of subclonal mutations for each patient that could be attributed to APOBEC activity are shown, with mutations in an APOBEC context shown in orange and all other mutational contexts shown in green.

APOBEC mutagenesis occurs independently within single cancer cells and viruses, often resulting in strand-coordinated hypermutations (sometimes referred to as kataegis [1]). Evidence for *A3B* upregulation has been found in over half of all cancers [3] (reviewed elsewhere [31]). Additionally, our groups and others have identified APOBEC activity as contributing to branched evolution and the acquisition of subclonal mutations later in the evolutionary course of lung adenocarcinoma, estrogen receptor (ER)-negative breast cancer, head and neck squamous cell carcinoma, and esophageal adenocarcinomas [11, 35, 36]. A recent analysis of the intratumour heterogeneity present in 100 TRACERx patients with untreated surgically resected primary non-small-cell lung cancer revealed a significant correlation between frequencies of APOBEC signature mutation and the overall number of subclonal mutations [9]. Furthermore, in 19 patients, subclonal driver events were detected as occurring in the APOBEC mutational context [9] (Figure 1A). Moreover, tumours with the largest number of subclonal mutations had extensive evidence of APOBEC mutational signatures, suggesting that APOBEC activity is a strong mutagenic force late in cancer evolution (Figure 1B and C). Subclonal mutations generated from APOBEC activity could potentially drive cancer evolution by enabling the acquisition of late driver mutations. Across multiple types of cancer, there is evidence that APOBEC mutagenesis is responsible for creating driver mutations [5, 8, 9, 12, 31]. The most striking examples are two helical domain hot spot mutations in *PIK3CA* in papillomavirus-positive head and neck squamous cell carcinomas (E542K and E545K) [8].

APOBEC expression has also been reported to impact responses to cancer therapy. In ER-positive breast cancer, *A3B* mRNA expression levels inversely correlated with clinical benefit to tamoxifen, and *A3B* overexpression correlated with tamoxifen resistance in mouse xenograft models, implicating the enzyme in promoting drug resistance [6]. Similarly, an enrichment of *A3B* mutations was observed in chemotherapy-resistant urothelial carcinomas [37].

**Figure 2.**
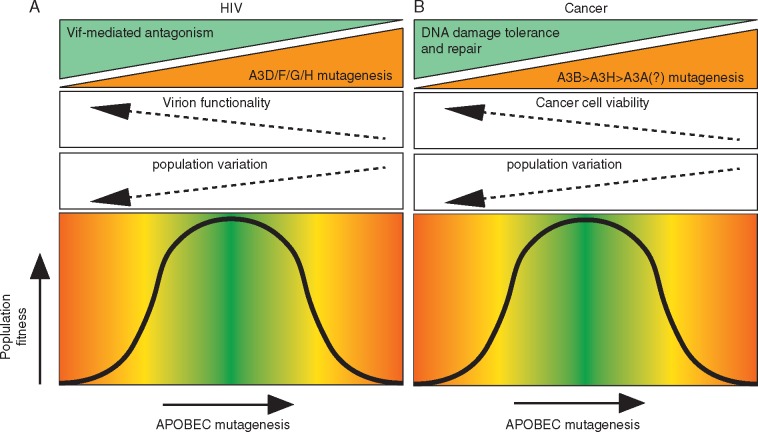
Parallels between APOBEC mutagenesis in HIV and cancer. (A) Within HIV, APOBEC mutagenesis is counteracted through Vif. As APOBEC mutagenesis increases, the chance of lethal mutagenesis and population variation increases. The trade-off between lethal mutagenesis and population variation creates an optimal range in which APOBEC mutagenesis increases population fitness. (B) Within cancer, the toxic effects of APOBEC mutagenesis are counteracted through DNA damage repair and DNA damage tolerance. As APOBEC mutagenesis increases, the chance of lethal mutagenesis and population variation increases. The trade-off between lethal mutagenesis and population variation creates an optimal range in which APOBEC mutagenesis increases population fitness.

APOBEC-induced mutagenesis is also prevalent in HIV-1 proviral DNA sequences [38], with some reports estimating the percentage of new mutations attributable to APOBEC activity to be as high as 98% [39]. However, other studies have estimated lower contributions for APOBEC in HIV-1 genetic variation, and definitive work has yet to be done to dissociate the contributions of APOBEC from mistakes made by the non-proofreading viral reverse transcriptase [38, 40]. Nevertheless, APOBEC activity has been linked to early diversification in newly infected individuals as the transmitted founder virus adapts in response to the immune pressure exerted within its new host [41]. Thus, APOBEC mutagenesis has the capability to increase subclonal diversity in both cancer and HIV, with the potential for a profound impact on the subsequent evolution of both the tumour and the virus (Figure 2).

The fact that APOBEC increases diversity is likely to be important for the clinical course of both diseases, since genetic heterogeneity is a substrate for Darwinian evolution and is likely to confound treatments. Indeed, in some cancers, increased intratumour heterogeneity has been shown to correlate with a shorter progression free survival [42, 43]. Additionally, measures of genetic heterogeneity have been associated with poor progression free survival in cancer [44] and intermediate thresholds of copy number instability correlate with poorest clinical outcomes [31], as they presumably allow for diversity without exceeding toxic levels of genomic instability (Figure 2). Paralleling findings in cancer, among patients with HIV exhibiting rapid disease progression, there are fewer hypermutated viral sequences as well as fewer minimally edited sequences, suggesting that suboptimal APOBEC activity may enhance genetic diversity, allowing an increase in pathogenicity [39] (Figure 2). From a clinical standpoint, this suggests that successful and complete inhibition of APOBEC enzymatic activity may provide a way to limit subclone diversification and potentially enable normal adaptive immune responses to control or clear HIV-1 infection [45]. Conceivably, complete inhibition of APOBEC enzymatic activity may limit tumour evolution and boost the efficacy of a variety of targeted or immune therapies that often fail due to the acquisition of escape variants brought about by resistance mutations and/or subclonal neoantigens.

## Additional lessons from HIV: APOBEC mutagenesis fuels drug resistance and immune escape

In order for HIV and cancer to replicate effectively in human hosts, these pathogenic entities must constantly avoid detection and destruction by innate and adaptive immune responses. As a consequence of this predator–prey relationship, lentiviruses and cancer have evolved elegant mechanisms to avoid or actively counteract immune responses. One of these overlapping mechanisms between HIV and cancer appears to entail co-opting APOBEC mutagenesis as a means of immune evasion.

**Figure 3.**
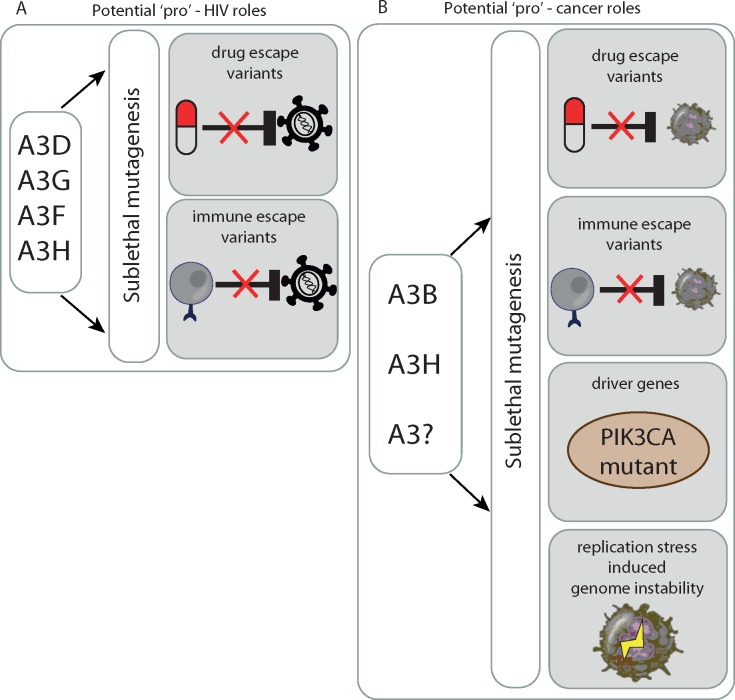
Potential roles of APOBEC supporting the escape and progression of HIV and cancer. (A) Sublethal APOBEC mutagenesis promotes the formation of drug escape and immune escape variants in HIV that will be selected upon exposure to treatment or the immune system. (B) Sublethal APOBEC mutagenesis promotes the formation of drug escape and immune escape variants in cancer that will be selected upon exposure to treatment or the immune system. APOBEC mutagenesis also underlies the formation of driver gene mutations and potentially also replication stress-induced genomic instability, although the latter is speculative.

**Figure 4.**
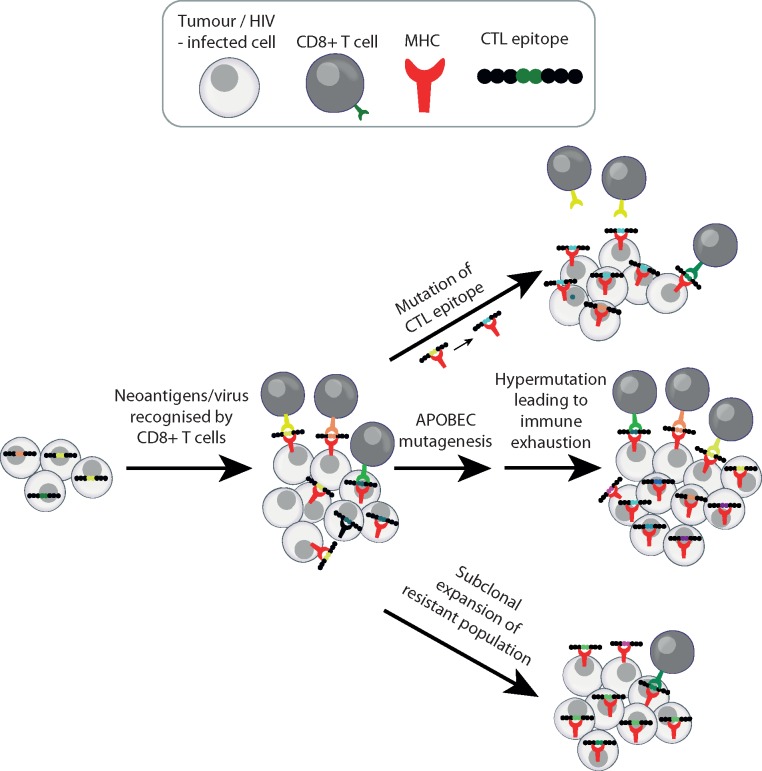
APOBEC mutagenesis promotes HIV immune escape and potentially also that of cancer. APOBEC mutations have been identified in cytotoxic T lymphocyte epitopes of HIV. APOBEC hypermutation has been linked to PD-L1 ligand overexpression and potentially contributes to immune exhaustion of tumour infiltrating lymphocytes. Studies within the HIV literature are suggestive of sublethal APOBEC mutagenesis driving the expansion of escape variants.

Lentiviruses and host A3 genes have co-evolved for millions of years in non-human primate relatives [46] and more recently in humans [47]. HIV-1, as well as other lentiviruses, has adapted to suppress A3-mediated innate anti-viral activity through utilizing the viral infectivity factor (Vif) as a defensive measure [15, 25]. Vif counteracts A3 functionality by polyubiquitination and proteasomal degradation [48]. Additionally, Vif has been shown to affect A3G translation through mRNA binding [49] and *A3G* gene transcription through heterodimerization with the transcription co-factor CBF-β [50]. These discoveries indicate that targeting the Vif-APOBEC3 interaction may be a novel avenue to combat HIV infection. However, over a decade of virology research into the A3-mediated restriction mechanism has demonstrated that interventions in this pathway may be a double-edged sword. Several studies have found that the range of APOBEC mutagenesis within HIV virions can vary largely. Lethal HIV mutagenesis will be selected against *in vivo*, and moderate APOBEC mutagenesis appears to induce sublethal variation that fuels viral heterogeneity and immune escape [41, 51–53] (Figure 3). Similar to cancer [54], viral genetic heterogeneity together with an appropriate selective pressure can enable the emergence of resistant populations. Sublethal G→A mutations in the DNA sequence motif preferred by A3G have been observed in drug-resistant HIV variants [55–58] and immune escape variants [38, 41, 51, 53] (Figure 2). Mutations in the APOBEC motif are enriched in cytotoxic T lymphocyte epitopes of HIV, and can result in diminished CD8+T cell responses against previously antigenic epitopes, suggestive of selection as a consequence of immune escape [38] (Figure 4). Finally, within cancer there is early evidence that APOBEC correlates with the overexpression of the immune checkpoint molecule PD-L1, potentially contributing to the development of immune exhaustion [59] (Figure 4). Indeed, early clinical trial data suggest that reversing immune exhaustion with an anti-PD-L1 antibody enhances the immune response against HIV in a subset of participants [60].

Some of the evolutionary dynamics that have been identified in HIV in response to therapy resistance also appear to apply to cancer. For example, in HIV-1, the V3 loop of gp120 interacts with the host cellular coreceptor CCR5 in order to gain entry to the cell. CCR5 antagonists bind CCR5 and prevent the entry of CCR5-tropic HIV-1 [61]. A common route of CCR5 antagonist resistance is the emergence of HIV-1 variants using CXCR4 instead of CCR5 as a coreceptor for cellular entry [62, 63]. Independent studies detected minor CCR5 antagonist-resistant variants containing resistance mutations in the APOBEC context at baseline that were rapidly selected through therapeutic selective pressure [55, 56]. Complementary to these *in vivo* studies, *in vitro* experiments point towards APOBEC mutagenesis generating subclonal resistance mutations, which are under positive selection during drug exposure [58]. Similarly, in cancer, minor drug resistant variants are rapidly selected upon treatment (reviewed in Schmitt et al. [64]), although the contribution of APOBEC in this process remains to be quantified.

An optimal range of APOBEC mutagenesis may exist for both HIV and tumour evolution (Figure 2). It has been shown that several Vif mutants are less potent in inhibiting A3G, with Vif-K22H incapable of fully neutralizing A3G and therefore appearing to enable sufficient levels of sublethal A3G mutagenesis for the emergence of antiretroviral resistance [57]. Thus, it appears that suboptimal A3G mutagenesis is less effective in inactivating the viral genome and in contrast may promote drug resistance and immune escape [38, 45, 58]. There are also parallels in the context of APOBEC mutagenesis of the cellular genome. Extensive A3B and A3A mutagenesis induces a DNA damage response (DDR) and at excessive levels it is toxic to the cell [2, 65–69]. Therefore, similar to HIV, cancer cells may have to attenuate APOBEC mutagenesis, enhance repair mechanisms, and/or dampen the DDR pathways to help ensure optimal cell survival (Figure 2). For instance, the loss of p53 enables DNA damage tolerance to A3B-mediated mutagenesis [65] and correlates positively with A3B overexpression in breast cancer [2]. Furthermore, a recent report focusing on Y-family polymerases and PrimPol, a translesion synthesis polymerase with re-priming properties, found that PrimPol as well as POLK and POLI may serve to limit the detrimental effects of APOBEC mutagenesis [70].

**Figure 5.**
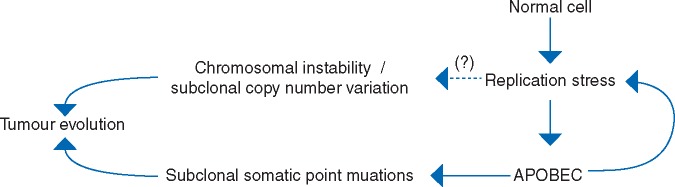
A feedforward replication stress-APOBEC loop potentially drives subclonal somatic point mutations and copy number variations. APOBEC induces subclonal somatic point mutations and has recently been shown to induce replication stress. APOBEC-mediated replication stress could potentially contribute to CIN.

In addition to sublethal APOBEC mutagenesis driving tumour heterogeneity, APOBEC has recently also been shown to produce replication stress in cancer [65, 71, 72]. This complements our previous observation that replication stress itself can drive A3B expression and activity [73], which suggests a positive feedforward loop involving replication stress and APOBEC mutagenesis (Figure 5). We have previously implicated replication stress in inducing a widespread endogenous DDR as a biological barrier to tumour progression [74–76], as well as driving chromosome segregation errors and ensuing chromosomal instability (CIN) [76, 77]. It is therefore conceivable that oncogene-induced and APOBEC-induced replication stress itself is a driver of CIN in cancer. Furthermore, APOBEC may provide a cancer cell the necessary genomic plasticity to evade immune surveillance. Firstly, a large subclonal neoantigen burden has been associated with a poor response to immunotherapy [78] and conceivably APOBEC-induced subclonal neoantigens may contribute to this process. Although the mechanism of how subclonal neoantigens potentially confound the response to immunotherapy is still unclear, it is conceivable that subclonal neoantigens foster the outgrowth of T cells that are reactive towards subclonal neoantigens that outcompete T cells reactive towards clonal neoantigens. This may result in the outgrowth of T cells that are only reactive to a proportion of the tumour population, diminishing chances of total tumour control. Secondly, ongoing chromosome missegregations (i.e. CIN) may enable the dynamic loss of immunogenic clonal neoantigens [79]. In effect, CIN-mediated loss of clonal neoantigens (provided these mutations are not required for tumour fitness) as well as the acquisition of subclonal neoantigens (due to APOBEC activity) could both aid immune evasion. Interestingly, highly aneuploid (i.e. an abnormal state of chromosomal copy number) tumours have less immune infiltration and are associated with a worse patient survival than less aneuploid tumours [80, 81]. It is yet to be quantified to which extent APOBEC-induced replication stress and potential ensuing CIN contributes to the process of immune evasion.

## Therapeutic considerations

By analogy to hypermutation and hypomutation within HIV [45], there are at least two general strategies for targeting APOBEC in cancer. The first strategy is therapy by hypermutation, by enhancing the mutagenic effects of APOBEC to the point where cancer cells suffer catastrophic levels of DNA damage and selectively die. Indeed, recent studies have suggested that DDR inhibitors, such as PARP and ATR inhibitors, may sensitize tumour cells with high levels of APOBEC to an APOBEC-dependent death [65, 71, 72].

The second strategy is therapy by hypomutation, by inhibiting APOBEC-dependent tumour evolution and potentially suppressing adverse outcomes including recurrence, metastasis, and drug resistance. Constraining cancer evolvability may be accomplished with drugs to inhibit APOBEC gene expression [73, 82] or with chemical inhibitors of DNA deaminase activity (for exemplary studies on A3G see [83–85]). Proof of principle has been achieved with a genetic knockdown of *A3B* causing an improvement in the durability of tamoxifen treatment of ER+ xenograft tumours in mice [6].

Besides harnessing APOBEC through a cancer cell intrinsic mechanism, a complementary strategy may be provoking immune responses to cancer cells with high levels of APOBEC-induced neoantigens. It is important to consider whether neoantigens are present in the trunk of the tumour (clonal) or in the branches (subclonal), since clonal neoantigens may improve response to immune checkpoint blockade in contrast to subclonal neoantigens [78]. Interestingly, one APOBEC-mediated mutational signature (signature 2) is primarily found in the branches of multiple different cancer types [12]. In contrast, the other APOBEC-mediated mutational signature (signature 13) is primarily found in the trunk of bladder cancer [12]. This could potentially explain paradoxical effects of APOBEC mutagenesis on immune surveillance and patient outcome. For example, in breast cancer, A3B expression is reported to worsen overall patient survival [6, 7]. In contrast, APOBEC mutagenesis within bladder cancer has been correlated with an improved patient outcome [86, 87], although the influence of APOBEC on survival in bladder cancer is still a matter of debate [88]. Therefore, it is conceivable that patients with tumours containing extensive clonal APOBEC mutagenesis, in contrast to subclonal APOBEC mutagenesis, are more suitable for immune checkpoint blockade.

It is also important to note that several APOBEC family members including *A3G* and *A3H* are highly expressed in immune cells, including tumour infiltrating T cells, and correlated with improved outcomes [89, 90]. Thus, as studies advance, it will be crucial to not only consider APOBEC expression and mutation signature in the tumour itself, but also APOBEC expression in the larger tumour microenvironment. Immunohistochemistry approaches would be ideal to help address these relationships, but specific monoclonal antibodies for each human APOBEC family member have been challenging to develop due to extensive protein similarity.

## Discussion

### Conclusions

Despite the numerous studies that have detected APOBEC-associated mutations by sequencing clinical cancer samples, it is still not fully clear why APOBEC mutagenesis is such a widespread and recurring mutational signature. In this review, we have postulated functions of APOBEC in cancer through exploring the past virology research focused on HIV and APOBEC. Within the field of virology, sublethal APOBEC mutagenesis of HIV virions has been linked to increasing HIV diversity and the creation of drug and immune escape variants. Conceivably, similar functions for APOBEC mutagenesis may be operating, and be selected for, during cancer evolution with clear opportunities for the development of novel therapeutic interventions.

## Funding

This work was partially funded from Cancer Research UK, Rosetrees and the University College London Hospitals Biomedical Research Centre (NM); 

the Danish Cancer Society, Swedish Research Council, NovoNordisk Foundation (ID 16584), Danish National Research Foundation (project CARD) and Danish Council for Independent Research (JB); 

IAVI with the generous support of USAID and other donors (JH) (a full list of IAVI donors is available at www.iavi.org); grants from the National Institutes of Health (NIAID R37 AI064046 and NCI R21 CA206309 (RSH). 

This work was supported by Cancer Research UK (TRACERx), the Rosetrees Trust, NovoNordisk Foundation (ID 16584), EU FP7 (projects PREDICT and RESPONSIFY, ID number 259303), the Prostate Cancer Foundation, the Breast Cancer Research Foundation, the European Research Council (THESEUS), and National Institute for Health Research University College London Hospitals Biomedical Research Centre (CS). The contents of this manuscript are the responsibility of the authors and do not necessarily reflect the views of USAID or the US Government. RSH is the Margaret Harvey Schering Land Grant Chair for Cancer Research, a Distinguished McKnight University Professor, and an Investigator of the Howard Hughes Medical Institute.

This work was supported by the Francis Crick Institute which receives its core funding from Cancer Research UK (FC001169), the UK Medical Research Council (FC001169), and the Wellcome Trust (FC001169).

## Disclosure

RSH is a co-founder, consultant, and shareholder of ApoGen Biotechnologies Inc. 

CS is a member of the advisory board of ApoGen Biotechnologies Inc. All remaining authors have declared no conflicts of interest.
